# The British Medical Association

**Published:** 1837-01

**Authors:** 


### THE BRITISH MEDICAL ASSOCIATION.

On Thursday, the 27th October, a meeting of medical practitioners was held, pursuant to public notice, at the Bridge-House Hotel, Southwark, for the purpose of establishing a new medical society. The meeting was numerously attended. George Webster, Esq., of Dulwich, was in the chair; and, in the course of his opening speech, he detailed the plan upon which it was proposed to found the society, and its nature and objects. The following propositions contain a general outline of the views and intentions of the gentlemen who have taken a lead in this measure.

"It is proposed, 1st. That the medical general practitioners of England and Wales shall form themselves into an Association, for the purpose of exciting and cherishing kindly and honorable feelings towards each other, and of guarding, watching over, and protecting the rights, privileges, interests, and respectability of the profession.

"2dly. That the society should be called 'The British Medical Association.'

"3dly. That those gentlemen willing to become members, shall, at an early meeting, appoint proper officers: such as a President, Vice-President, Secretaries, Councillors, Committees, &c., and shall form a code of laws for the government of the Association, to be submitted to the consideration of a subsequent general meeting.

"4thly. That the Association shall hold frequent meetings for the transaction of business.

"5thly. That it shall oppose all encroachments from without, and all dishonorable or unprofessional conduct among its members.

"6thly. That it shall, by all legal means, or by application to parliament, if considered necessary, endeavour to remove all professional grievances, evils, and hardships.

"7thly. That it shall protect its members from all illegal or unjust prosecutions.

"8thly. That it shall endeavour to form a benevolent fund for the assistance of decayed members of the profession, and for the benefit of their widows and orphans.

"9thly. That, to effect these important purposes, subscriptions shall be paid by the members, in such manner as shall be hereafter agreed upon, and donations requested from their friends and the profession at large.

"10thly. That the Association shall endeavour to extend its expected advantages over the kingdom, by corresponding with, and inviting the co-operation of, their medical brethren in cities, towns, or local districts; and by recommending them to form themselves into societies, having the same or similar important ends and objects in view.

"11thly. That the members and their friends shall dine together at least once a year, in the metropolis; and that the Association shall, by all means in its power, endeavour to promote the welfare, prosperity, and union of its own body in particular, and uphold the dignity, respectability, and usefulness of the whole medical profession."

It is impossible to object to any thing contained in these propositions; but, as the great body of provincial practitioners are already united in a most respectable and very flourishing society having similar objects, we may be allowed to doubt whether a less comprehensive title might not be more suitable. We wish all success to the new Institution.

